# X-chromosome-linked miR548am-5p is a key regulator of sex disparity in the susceptibility to mitochondria-mediated apoptosis

**DOI:** 10.1038/s41419-019-1888-3

**Published:** 2019-09-11

**Authors:** Paola Matarrese, Paolo Tieri, Simona Anticoli, Barbara Ascione, Maria Conte, Claudio Franceschi, Walter Malorni, Stefano Salvioli, Anna Ruggieri

**Affiliations:** 1 Center for Gender Specific Medicine, Istituto Superiore di Sanità, viale Regina Elena 299, Rome, Italy; 2 CNR National Research Council, IAC Institute for Applied Computing, Via dei Taurini 19, Rome, Italy; 3 Data Science Program, La Sapienza University of Rome, Rome, Italy; 4 Department of Experimental, Diagnostic and Specialty Medicine (DIMES), University of Bologna, Bologna, Italy; 5 Interdepartmental Centre “L. Galvani” (CIG), University of Bologna, via San Giacomo 12, 40126 Bologna, Italy; 6 IRCCS Istituto delle Scienze Neurologiche di Bologna, Via Altura 3, 40139 Bologna, Italy; 7 Lobachevsky State University of Nizhny Novgorod, Nizhny Novgorod, Russia; 8 School of Mathematical, Physical and Natural Sciences and Faculty of Medicine, University of Tor Vergata, Rome, Italy

**Keywords:** Apoptosis, Genetics research

## Abstract

Sex dimorphism in cell response to stress has previously been investigated by different research groups. This dimorphism could be at least in part accounted for by sex-biased expression of regulatory elements such as microRNAs (miRs). In order to spot previously unknown miR expression differences we took advantage of prior knowledge on specialized databases to identify X chromosome-encoded miRs potentially escaping X chromosome inactivation (XCI). MiR-548am-5p emerged as potentially XCI escaper and was experimentally verified to be significantly up-regulated in human XX primary dermal fibroblasts (DFs) compared to XY ones. Accordingly, miR-548am-5p target mRNAs, e.g. the transcript for Bax, was differently modulated in XX and XY DFs. Functional analyses indicated that XY DFs were more prone to mitochondria-mediated apoptosis than XX ones. Experimentally induced overexpression of miR548am-5p in XY cells by lentivirus vector transduction decreased apoptosis susceptibility, whereas its down-regulation in XX cells enhanced apoptosis susceptibility. These data indicate that this approach could be used to identify previously unreported sex-biased differences in miR expression and that a miR identified with this approach, miR548am-5p, can account for sex-dependent differences observed in the susceptibility to mitochondrial apoptosis of human DFs.

## Introduction

A number of diseases have been documented to show a different incidence, progression, and even response to therapy between women and men. Among these are cardiovascular, cancer, metabolic, infectious, or immune diseases^1–5^. However, albeit the impact of chromosomal differences has been suggested, relatively few studies have investigated whether sex chromosomes could play a role^6^. In fact, also the association analyses conducted with more recent DNA sequencing tools have very often neglected to consider the influence of sex, i.e. the X and Y chromosomes, on gene expression^7,8^. The importance of such a field of investigation resides on the idea that the understanding of the mechanisms underlying gender disparity could help in our knowledge of the pathogenetic mechanisms as well as in the development of the so-called precision medicine^9–11^. Some insights are derived from studies carried out at cellular level, as suggested by NIH as necessary step to fill the gap^12,13^. Differences in the behavior of cells from males and females have been investigated in the recent years as concerns their homeostasis. In particular, a series of studies have been carried out with the aim of deciphering whether a disparity in the response to stress could take place in cells coming from males and females. XX and XY cells, independently from their origin (e.g. mice, rats, and humans) and histotype (e.g. muscular and endothelial vessel cells, cardiomyocytes, neurons, fibroblasts), display different responses to stress: XX cells are more prone to autophagic cytoprotection and senescence, whereas XY cells easier undergo apoptosis^14–17^. Hence, as a general rule, under the same stress, e.g. oxidative, a sex disparity can be found in a series of intracellular parameters of importance in cell fate.

On these bases the idea that “cell sex” could determine the answer of a cell type to altered environmental conditions and become a determinant in disease onset or progression and in response to therapy has been proposed by recent studies^18^. This matter belongs to the so-called gender medicine, a field of investigation that encompasses several areas of research including immunity/autoimmunity, cardiovascular diseases, neurodegeneration, and cancer^19–22^. In all these pathologic conditions cell homeostasis (of lymphocytes, vessel or cardiac cells, neuronal cells, cancer cells), i.e. the control of cell survival or death, is critical. Hence, the discovery of sex-based differences in cell’ response to exogenous stressing agents could possibly provide clues on both the pathogenic mechanisms of diseases displaying a gender disparity and on the different response to therapy that has been observed^9^.

Evidence is accumulating for a sex-biased expression of regulatory elements such as microRNA (miRs) across a variety of tissues that could have functional implications^23,24^. The reasons for such differences are at present not totally clear. One straight-forward hypothesis is that some of them are encoded in sex chromosomes. To date, only two miRs are reported to be present in the Y chromosome (miR-3690 and miR-6089), and both are also present in the X chromosome (NCBI gene database).

On the contrary, the X chromosome is a rather populated one, and contains about 10% of the total miRs^25^. According to the rule of gene dosage, the number of genes active in each cell should be the same, no matter whether it is XX or XY, due to the fact that one of the two X chromosomes is inactivated in female’ cells. However, it is well known that a number of X-linked genes escape X chromosome inactivation (XCI)^26–28^. Recently, it has been shown that an incomplete XCI affects 23% of X chromosome genes^29^. A systematic bioinformatic analysis of these genes has not been performed, due to the extremely heterogeneous scenario of XCI (escaper genes may display different levels of expression between tissues and subjects)^30^. However, incomplete XCI has been proposed as a mechanism that can introduce phenotypic diversity^31,32^. In the same vein, it has been proposed that some of the X-linked miRs may escape XCI as well, and can be differently expressed in XX and XY cells, thus accounting (at least partially) for the observed sex-dependent differences in immune and stress responses^25^. In particular, some miRs are actually coded within the body of escaper genes and are therefore likely to be regulated in the same way.

We therefore performed an extensive data exploration via specialised and cross-linked databases to identify miRs differentially expressed between XX and XY cells that can account for cell-sex disparity. In particular, (i) we identified genes that always appear to escape XCI in many different tissues; (ii) we investigated the presence of miRs within the region of such genes; (iii) we extracted from databases the experimentally validated targets of these miRs and performed a pathway enrichment; (iv) we experimentally tested the results by checking with quantitative real-time PCR the expression of selected target genes, as well as their biologic functional effects on cell fate; and finally (v) performed overexpression and silencing experiments of the identified miR (548am-5p) to validate its possible role in sex-specific phenotypic difference.

## Materials and methods

### Selection of XCI escaper genes from available datasets

We used two main data sources about genes escaping X chromosome inactivation (XCI), hereinafter XCI escaper genes: the analyses of Carrel and Willard^26^ and of Cotton et al.^30^.

In the above-mentioned papers, genes are defined as follows. According to the work of Carrel and Willard^26^ and its Supplementary Table S3, genes escaping X inactivation are expressed in at least 7 out of the 9 hybrid cell lines tested (genes expressed in 0 to 2 out of 9 hybrid lines tested are X inactivated; genes expressed in 3 to 6 out of 9 hybrid lines tested are subject to variable escape).

In the study of Cotton et al.^30^, specifically in the Additional file 7, Table S5, genes are defined escapers based on the percentage of inactive X chromosome expression as a ratio of active X chromosome expression.

According to the above definitions and data, we found 99 escaper genes IDs in the analyses of Carrel and Willard.^26^ (expressed in ≥7 of the 9 lines tested, hereinafter Carrel dataset, Table S1), which mapped to 64 valid gene name IDs after cross-check on HGNC Multi-symbol checker^33^, and 68 escaper genes IDs in Cotton et al., which mapped to 65 valid gene name IDs after check on HGNC (hereinafter Cotton dataset, Table S2). Twenty-three escaper genes are shared between the two datasets (intersection), while 106 escaper genes appear at least in one of the two datasets (union; Table S3).

### Identification of X-linked miRs putatively escaping XCI

Analysis performed on the 106 genes defined as XCI escaper in either the Carrell dataset or the Cotton dataset identified 6 genes that contain 12 miRs in their chromosomic loci (Table 1), according to the host gene-miRNA mapping services. To do this, we mapped escaper genes (Uniprot AC) to transcripts (Ensembl Transcript ID, ENST) by using the Uniprot Knowledgebase mapping services^34^ (Table S4), and the X chromosome miR IDs to the host gene transcripts IDs (Ensemble Transcript ID) by using the specific services provided by the databases mirWalk 2.0 (ref. ^35^) and miRBase^36^ (Table S5). The database miRBase provides 118 *Homo sapiens* miRNAs on chromosome X; from these IDs, we obtained the miR IDs to Ensembl Transcript IDs map by using the “host gene” mapping service provided by the database mirWalk 2.0. (Table S5**)**. We finally obtained the list of escaper genes hosting an miR in their locus (Table S6).

**Table 1:** Escaper genes hosting an miRNA in their locus

Gene	miRNA
CSF2RA	hsa-miR-3690
**CTPS2**	**hsa-miR-548am**
GABRE	hsa-miR-224
GABRE	hsa-miR-452
HTR2C	hsa-miR-1264
HTR2C	hsa-miR-1298
HTR2C	hsa-miR-1911
HTR2C	hsa-miR-1912
HTR2C	hsa-miR-448
HTR2C	hsa-miR-764
*PUDP*	*hsa-miR-4767*
VGLL1	hsa-miR-934

Gene names, alphabetical order; black: escaper in Cotton et al.^30^ (2013); bold: escaper in Carrel and Willard^26^; italics: escaper in both datasets

Since for further biological validation experiments we decided to use primary dermal fibroblasts (DFs), we took advantage of the dataset by Tukiainen et al.^29^, in which the difference in the expression of 681 X -chromosome genes between males and females in 29 human tissues is reported. According to this study, three genes out of six of Table 1 are not expressed in fibroblasts (CSF2RA, HTR2C, VGLL1), one has a negative Log Fold Change (GABRE), suggesting that in these cells this gene is not really escaping XCI. The two remaining genes appear actually escapers in fibroblasts (CTPS2 and PUDP), so we focused on these two genes. CTPS2 gene has two miRs in its locus (miR-548am-5p and 548am-3p), while PUDP only one (miR-4767). From our previous data, XX and XY cells differ in terms of susceptibility to apoptosis^13,37,38^ thus we focused our attention on this aspect. According to the database Tarbase 8.0 (ref. ^39^), miR548am-5p has 332 experimentally validated target genes (Table S7). Pathway analysis carried out with the same knowledgebase via mirPath v.3 (ref. ^40^) on such 332 genes yielded six enriched pathways, among which apoptosis (Table S8). According to KEGG pathway analysis via mirPath, eight genes are involved in apoptosis (APAF1, ATM, BAX, CAPN2, PIK3R1, PRKAR2B, TNFRSF10B, and XIAP, *p* value = 0.01340305) (Tables S7 and S8). On the same database Tarbase 8.0, miR-4767 has only three validated target genes (Table S7). Therefore, miR548am-5p seemed a good candidate to explain the sex-specific difference in susceptibility to apoptosis and was therefore selected for further analyses. Two additional X chromosome miRs present in loci subject to XCI were also selected as negative controls: miR-23c and miR-548ax (this latter belonging to the same family of miR-548am-5p).

### Cell culture and treatments

Primary DF cultures were available at the bio-bank of our laboratory and were established from biopsies of sun-protected forearm skin according to standard culture methods. All the donors gave their informed consent before biopsy was performed. In total, 16 subjects were studied: 8 female donors (31.37 ± 9.47 years) and 8 male donors (30.25 ± 4.7 years). DFs cultures were established and grown-propagated in Dulbecco’s modified Eagle’s medium (DMEM) (Life technologies, Carlsbad, California, USA) containing 25 mM glucose supplemented with 10% fetal bovine serum (FBS) (Life technologies, Carlsbad, California, USA) at 37 °C in a humidified atmosphere of 5% CO_2_. In addition, the medium contained 100 U/ml penicillin, 100 μg/ml streptomycin (Life technologies, Carlsbad, California, USA), 4 mM glutamine, and 1 mM pyruvate. Apoptosis was induced by treating cells with cycloheximide (CHX, 25 μg/ml) for 2 h and with tumor necrosis factor-alpha (TNF-α, 100 ng/ml) for additional 18 h. As alternative apoptosis inducer, we also used Staurosporine (Sigma, 50 nM overnight). All the analyses were performed on cells between fourth and 12th passage of culture and at nearly 80% confluence. To note, to exclude that the observed differences between XX and XY DFs were due, at least in part, to the effect of the estrogens and/or testosterone present in the fetal calf serum, we conducted parallel evaluations also using charcodylated serum. The results obtained were completely overlapping (data not shown). Therefore, on these bases, the whole study was carried out by using non-charcodylated serum.

### Quantitative analysis of the selected microRNAs by TaqMan qRT-PCR

Total RNA, including miRs, was isolated from DFs using the miRvana Paris Kit (Thermo Fisher), according to the manufacturer’s instructions. RNA samples, after quality and quantity evaluation using a NanoDrop ND-1000 spectrophotometer, were stored at −80 °C until used in the experiments. Quantification of miR-23c, miR-548am-5p, miR-548ax, and RNU6B and RNU44 (the two latter as housekeeping miRs, were used for normalization) expression was carried out in triplicate using specific inventoried TaqMan MicroRNA Assays (Thermo Fisher), according to the manufacturer’s instructions. Briefly, 10 ng of RNA was retrotranscribed by the Taq-Man® MicroRNA Reverse Transcription (RT) Kit (Thermo Fisher) using individual miR-specific RT primers, and 1.3 μl of RT product was analyzed by quantitative real-time PCR (qRT-PCR) on the ABI7000 (Applied Biosystem). Threshold cycle (Ct) and baselines were determined by manual settings. miR expression was calculated by relative quantification and fold expression changes were determined by the 2^−ΔΔCt^ method, after normalization to the RNU6B and RNU44 ΔCt. 1.5 miRs fold changes between female and male cells were considered significant.

### Lentivirus production

The 293 GPR cells were used as HIV-1 packaging cells for lentivirus production. In these cells, gag-pol genes are expressed under control of an ecdysone-inducible promoter, so that the lentiviral particle production requires cell stimulation with the ecdysone analog ponasterone A (PonA). Lentivirus (LVS) were obtained by co-transfecting immediate-early CMV promoted vectors expressing the human pre-Mir-548am-5p or the anti-miR-548am-5p (SBI) and the VSV-G protein by Lipofectamine 2000 (Invitrogen). Transfected 293 GPR cells were induced 8 h post-transfection with 5 mM sodium butyrate and 2 μM of PonA. Twenty hours later, supernatants were replaced with fresh medium containing the inducers. LVS containing supernatants were finally harvested 24 and 48 h later, clarified, and concentrated by ultracentrifugation on 20% sucrose cushion 100,000 × *g*, 2 h at 4 °C. LVS preparations were titrated by measuring HIV-1 CAp24 contents by quantitative ELISA (Innogenetics, Gent, Belgium).

### Primary cells transduction

DFs from four male donors were transduced to overexpress miR-548am-5p and four female donors were transduced to downregulate miR-548am-5p. One hundred nanograms of HIV-1 CAp24 equivalents of LVS/10^5^ cells were adsorbed on DFs by spinoculation at 150 × *g* for 30 min at room temperature. Afterwards, cells were re-fed by adding fresh medium, incubated at 37 °C and harvested 48 h later. Parallel DF cultures were treated with 50 μM zidovudine (AZT), 30 min at 37 °C before transduction and used as controls for the specific lentivirus effect. Harvested cells were examined for transduction efficiency by flow cytometer analysis by quantification of GFP-positive cells. GFP-transduced cells were sorted to 99% purity by fluorescence-activated cell sorting (FACS) using a BD Biosciences cytometer (FACS Aria; BD Biosciences, San Jose, CA). Either GFP-negative or -positive cells were subsequently analyzed for miR-548am-5p level, Bax and Bcl-2 expression, and apoptotic susceptibility.

### Chemicals and reagents

All chemicals were the highest grade available from Sigma-Aldrich (St. Louis, MO, USA), unless otherwise indicated. 5,5,6,60-tetra-chloro-1,10,3,30-tetraethyl-benzimidazolyl-carbocyanineiodide (JC-1), tetramethylrhodamine (TMRM), monochlorobimane (MCB), and MitoSOX were all purchased from Life Technologies Corporation.

### Flow cytometry analyses

#### Mitochondrial membrane potential

The mitochondrial membrane potential (MMP) of DFs was studied by using JC-1 or TMRM (both Life Technologies Corporation). In line with this method, living cells were stained with 10 μM of JC-1 or with 1 μM TMRM.

#### Cell death

Quantitative evaluation of apoptosis was performed by a double staining flow cytometry method using FITC-conjugated Annexin V or APC-conjugated Annexin V (Life Technologies Corporation) and Trypan blue (TB) (Marine Biological Laboratory, MBL, Woods Hole, MA, USA) according to the manufacturer’s protocol.

#### Reacting Oxygen Species

Living cells were incubated in Hank’s balanced salt solution, pH 7.4, with dihydrorhodamine 123 (DHR123, Thermo Fisher) in polypropylene test tubes for 15 min at 37 °C (final concentration 10 μM). DHR123 dye freely diffuses into cells and is primarily oxidized by H_2_O_2_ producing green fluorescence.

#### Mitochondrial reacting oxygen species

Living cells (5 × 10^5^) were incubated with 5 μM MitoSOX (Red Mitochondrial Superoxide Indicator; Thermo Fisher, Eugene, OR) in complete medium, for 30 min at 37 °C. Cells were then washed in phosphate-buffered saline (PBS) and immediately analyzed on a cytometer.

#### Glutathione

Following the same protocol, glutathione (GSH) intracellular content was evaluated by using MCB (Thermo Fisher). Samples were washed twice in ice-cold PBS and immediately acquired by an LRS II cytometer (Becton and Dickinson, San Jose, CA, USA) equipped with a UVB laser.

### Quantitative evaluation of proteins

DFs were fixed with 4% paraformaldehyde (Carlo Erba, Milano, Italia) and then permeabilized by 0.5% Triton X-100 (Sigma-Aldrich). After washings, cells were incubated for 1 h at 4 °C with the following antibodies, alone or in combination, at a final concentration of 0.1 mg/ml: MAb to Bcl-2 (Santa Cruz Biotechnology, Inc., CA, USA); MAb anti-APAF-1 (Abcam, Cambridge, UK), PAb anti-BAX (Cell Signaling, New England Biolabs, UK), PAb anti-Bcl-xL (Santa Cruz), and MAb anti-XIAP (Cell Signaling) for 1 h at 4 °C. After washings, cells were incubated for 30 min at 37 °C with an anti-mouse antibody conjugated with Cy5 and/or with an anti-rabbit conjugated with PE (both Abcam). Cell samples were washed twice in PBS and immediately analyzed on a cytometer.

### Data and statistics

For flow cytometry studies, where not otherwise specified, samples were analyzed with a FACScalibur cytometer (BD Biosciences) equipped with a 488 argon laser and with a 635 red diode laser. At least 30,000 events were acquired. Data were recorded and statistically analyzed by a Macintosh computer using CellQuest software (BD Biosciences). The expression level of the analyzed proteins was expressed as median fluorescence. Collected data analysis was carried out by ANOVA two-way testing for repeated samples, using Graph Pad software (Graph Pad, San Diego, CA, USA). All data were verified in at least three independent experiments and are reported as means ± standard deviation (SD). Only values of *p* < 0.01 were considered as significant.

## Results

### miR-548am-5p is differentially expressed in XX and XY cells

Based on the bioinformatic analysis described in Materials and methods, miR-548am-5p resulted possibly expressed at different levels in XX and XY cells. Two additional miRs, miR23c and miR548ax, were considered as controls. These three miRs regulate a number of genes (663, listed in Table S5), in particular miR-548ax and miR-548am-5p regulate some genes that are involved in very important pathways affecting cell fate, such as p53 pathway, apoptosis, and protein ubiquitination (Table S6). To test whether the prediction of our in silico analysis was correct, we tested the expression of these three miRs by qRT-PCR in primary DFs from eight male and eight female subjects.

**Fig. 1:**
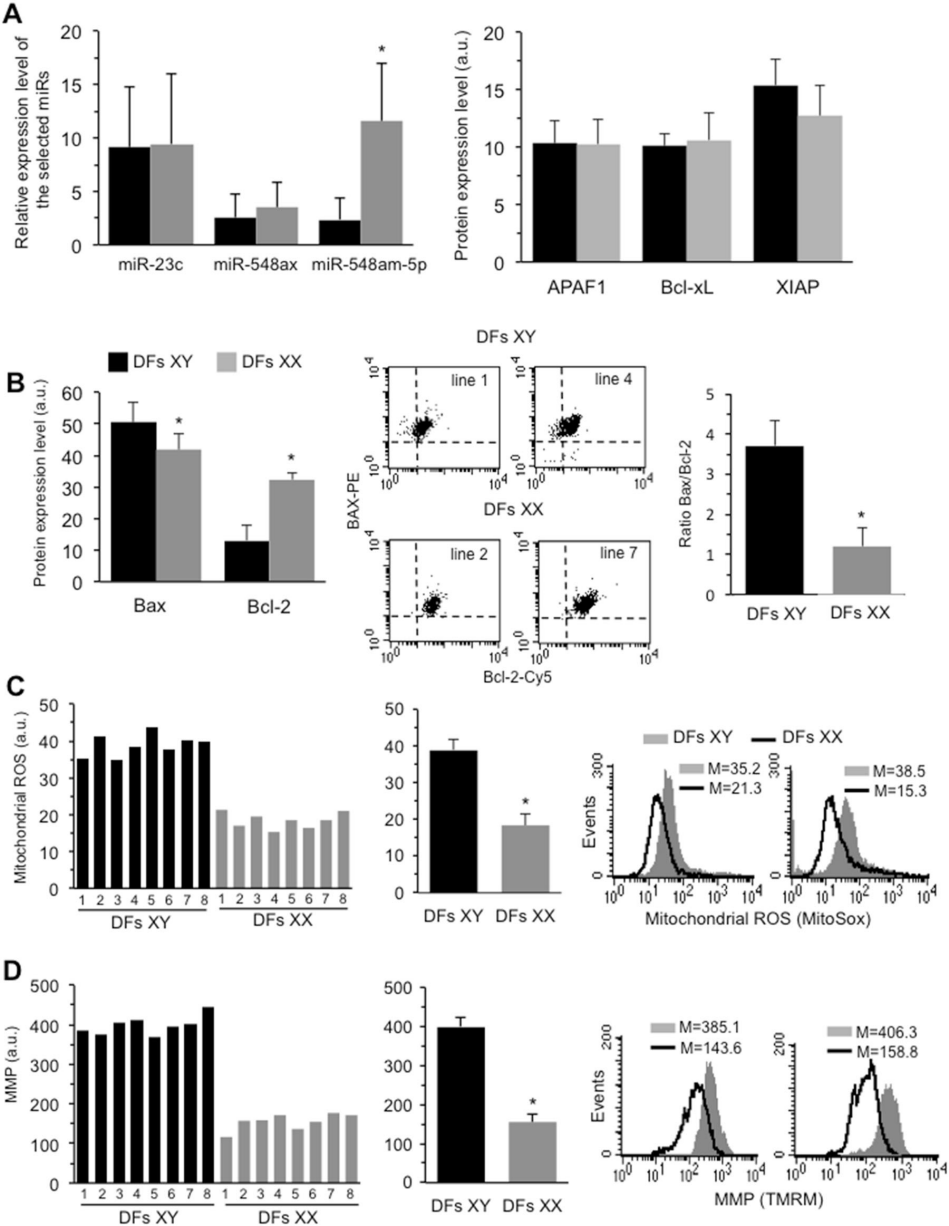
**a** Left panel: Expression level of the selected microRNAs in XX and XY DFs as quantitatively measured by qRT-PCR. The values of fold increase were calculated by the 2^−ΔΔCt^ method for each sample relative to the overall female or male samples mean. The fold increase values were obtained from eight female and eight male donors estimated in two independent quantitation reactions, with triplicate wells for each sample and for each microRNA. Right panel: Cytofluorimetric analysis of APAF1, Bcl-xL, and XIAP expression level in DFs isolated from eight male and eight female healthy donors obtained in three independent measures performed in each donor and reported as mean ± SD of the median fluorescence intensity. No statistically significant differences were evident between male and female DFs. **b** Left panel: Cytofluorimetric analysis of Bax and Bcl-2 expression level in DFs isolated from eight male and eight female (right panel) healthy donors obtained in three independent measures performed in each donor and reported as mean ± SD of the median fluorescence intensity. Central panel: Dot plots obtained in two representative XY and XX DFs after double cell staining with Bax and Bcl-2 specific antibodies. Right panel: Bar graph showing the ratio Bax/Bcl-2 obtained by pooling together measures acquired in DFs isolated from all the male or female donors. **c** Left panel: Bar graph showing a representative flow cytometric evaluation, performed by using MitoSOX-red, of mitochondrial ROS production in DFs isolated from eight male and eight female healthy donors. Central panel: Bar graph showing mitochondrial ROS production obtained by pooling together measures acquired in DFs isolated from all the male or female donors and reported as mean ± SD of the median fluorescence intensity. Right panels: Cytofluorimetric histograms of mitochondrial ROS evaluation obtained in two representative male and female DFs. **d** Left panel: Bar graph showing a representative flow cytometric evaluation, performed by using TMRM, of mitochondrial membrane potential (MMP) in DFs isolated from eight male and eight female healthy donors. Central panel: Bar graph showing MMP obtained by pooling together measures acquired in DFs isolated from all the male or female donors and reported as mean ± SD of the median fluorescence intensity. Right panels: Cytofluorimetric histograms of MMP evaluation obtained in two representative XY and XX DFs. Black columns XY DFs; gray columns XX DFs. *p < 0.01 between XY and XX DFs

The results shown in Fig. 1a (left panel) indicate that miR-548am-5p is the most differently expressed, being about fivefold overexpressed in XX DFs with respect to XY ones, whereas miR-23c and miR-548ax were equally expressed in both sexes. To further test whether this differential expression may have an effect on apoptosis susceptibility, we investigated the expression of specific target genes of miR548am-5p. Among these target genes, some apoptosis-related genes were predicted and experimentally validated, such as apoptotic peptidase activating factor 1 (APAF1), X-linked inhibitor of apoptosis protein (XIAP), B-cell lymphoma 2 (Bcl-2), Bcl-2-associated protein X (Bax), and Bcl-2-like 1 (Bcl-xL). Based on this, we first quantitated the levels of these proteins in XY and XX DFs. As shown in Fig. 1a (right panel), we did not observe any significant difference in the expression of APAF1, Bcl-xL, or XIAP between XY and XX DFs. In contrast, significant sex-related differences in the expression levels of Bax and Bcl-2 proteins were detected. In particular, in XY DFs, the Bax level was significantly higher and the Bcl-2 level was significantly lower (about threefold) than in XX ones. Consequently the Bax/Bcl-2 ratio was threefold higher in XY compared to XX DFs (Fig. 1b).

In addition, in XY DFs we also observed a significantly higher production of mitochondrial Reacting Oxygen Species (Fig. 1c), paralleled by a hyperpolarization of MMP(assessed using TMRM or JC-1) in comparison with XX DFs (Fig. 1d).

**Fig. 2:**
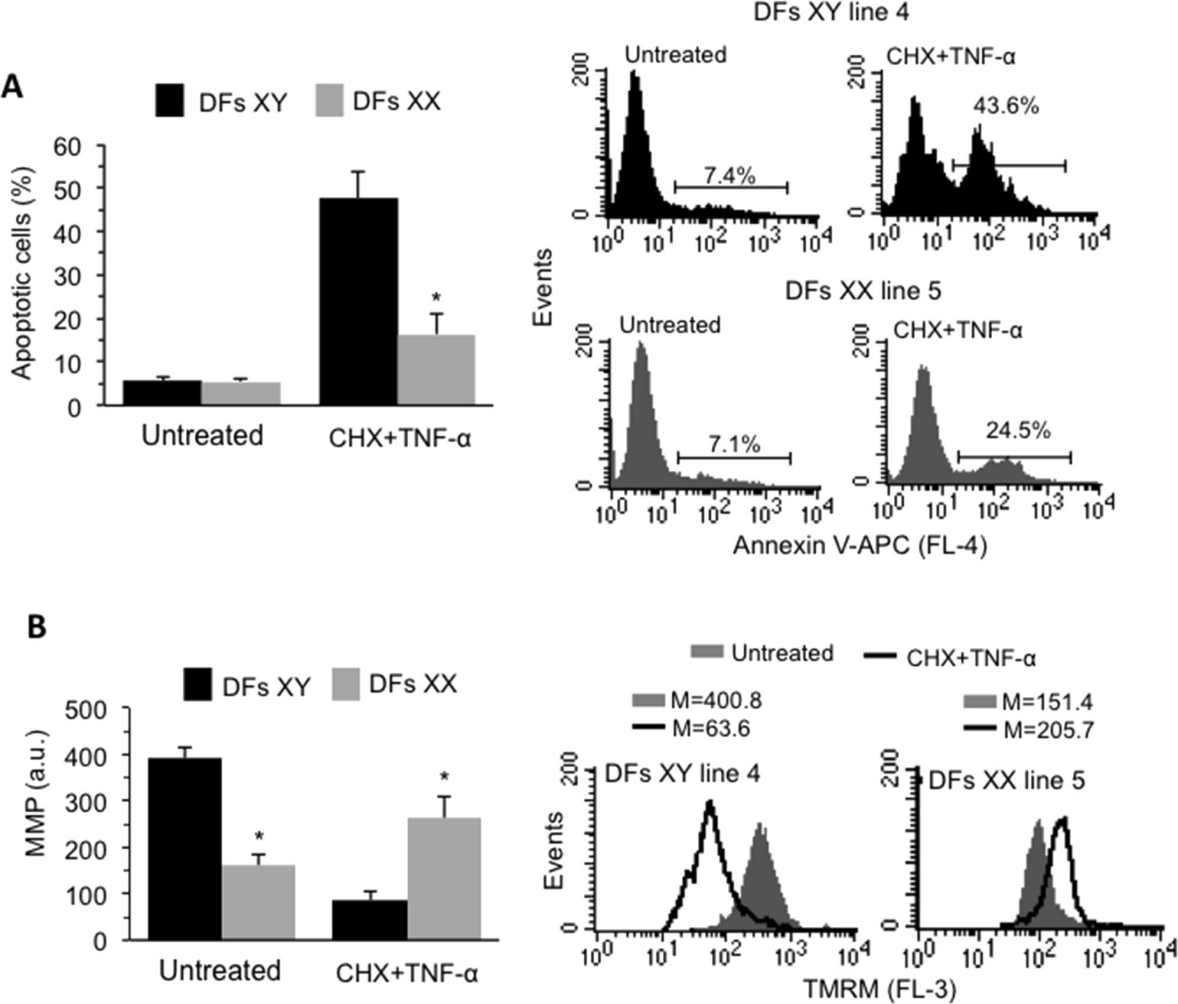
**a** Left panel: Bar graph showing flow cytometry analysis after staining with Annexin V-APC obtained in DFs isolated from eight male and eight female healthy donors reported as means ± SD. Right panel: Flow cytometry histograms of apoptosis evaluation obtained in one representative cell line of XY and XX DFs. Numbers represent the percentages of Annexin V-positive cells. **b** Left panel: Bar graph showing MMP obtained by pooling together measures acquired in DFs isolated from all the male or female donors and reported as mean ± SD of the median fluorescence intensity. Right panels: Cytofluorimetric histograms of MMP evaluation obtained in one representative cell line of XY and XY DFs. *p < 0.01 between XY and XX DFs

Anti-apoptotic proteins such as Bcl-2 bind and sequester pro-apoptotic proteins, including Bax, to regulate the mitochondrial pathway of apoptosis, by controlling the permeabilization of the outer mitochondrial membrane^41^. We therefore compared the apoptotic proneness of XY and XX DFs after treatment with cycloheximide (5 μM, CHX), an inhibitor of protein synthesis, and TNF-α (100 ng/ml), which trigger mitochondrial-dependent cell death pathway^42^. Following the pro-apoptotic treatment (CHX+TNF-α) for 18 h, about 50% of XY DFs and about 20% of XX DFs underwent apoptotic death (Fig. 2a). Accordingly, cytofluorimetric analysis showed a significant drop of MMP after treatment with CHX+TNF-α in XY but not XX DFs (Fig. 2b). Cell treatment with CHX or TNF-α alone was largely ineffective in inducing cell death, with no difference between XX and XY DFs. In particular, after CHX administration we found 7.1 ± 3.1% and 6.3 ± 2.3% of apoptotic cells in XY and XX DFs, respectively (*p* > 0.05 vs untreated cells), whereas TNF-α induced apoptosis in 9.2 ± 4.1% of XY DFs and in 6.7 ± 3.3 of XX DFs. Interestingly, a similar sex difference was found when a different apoptotic stimulation was used, i.e. 50 nM Staurosporine, overnight (XY DFs 21.4 ± 7.6; XX DFs 9.7 ± 5.4; XY vs XX DFs, *p* < 0.01) (data not shown).

Collectively these results indicated that male derived DFs were more prone to mitochondria-mediated apoptosis than female ones.

### Effects of overexpression of miR548am-5p in XY DFs

**Fig. 3:**
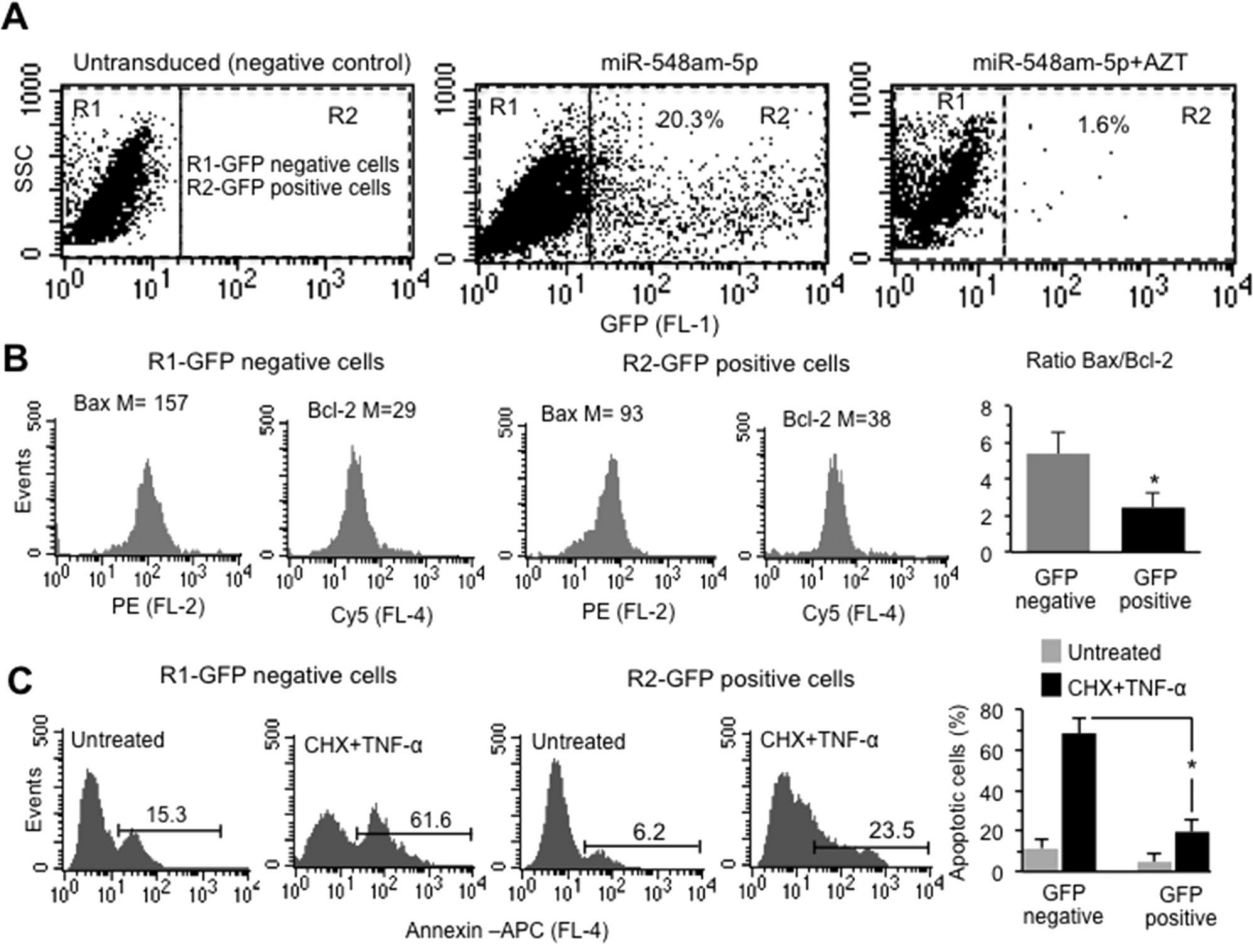
**a** Side scatter vs FL1 (GFP) dot plots obtained in one representative XY DFs cell line untransduced or transduced with GFP-miR548am-5p in the presence or absence of AZT. Numbers indicate the percentage of GFP-positive cells. **b** Cytofluorimetric histograms of Bax and Bcl-2 expression level in GFP-negative and -positive cells obtained in one representative XY DFs cell line after transduction with GFP-miR548am-5p. Numbers indicate the median fluorescence intensity. Bar graph on the right shows the ratio Bax/Bcl-2 of four XY DFs cell lines. *p < 0.01 between GFP-positive and GFP-negative cell populations. **c** Flow cytometry histograms of apoptosis evaluation in GFP-positive or -negative cell populations of one representative XY DFs cell line after transduction with GFP-miR548am-5p treated or not with CHX+TNF-α. Numbers represent the percentages of annexin V-positive cells. Bar graph showing flow cytometry analysis after cell staining with Annexin V-APC performed in triplicate and reported as means ± SD of four XY DFs cell lines

In order to verify whether the above-mentioned biological differences could be due to the different levels of miR548am-5p, we overexpressed this miR in DFs isolated from four different male subjects, by transduction with GFP-miR548am-5p-carrying lentivirus. Forty-eight hours after transduction, GFP-positive cells were sorted by FACS and level of miR548am-5p was determined by qRT-PCR. An average of 24.2 ± 5.3% of transduced GFP-positive cells was obtained (Fig. 3a, central panel, Fig. S1A). Upon transduction, the miR548am-5p level increased by almost four times with respect to the GFP-negative controls and by 1.7-fold compared to the mock-transduced cells (Fig. S1B). The significant difference in percentage of transduced cells (2 ± 0.43%) found in DFs treated with 50 μM of the inhibitor of lentivirus replication, AZT, indicated that miR548am-5p effect in male cultures was specific (Fig. 3a, right panel, Fig. S1A). In parallel, we evaluated by flow cytometry the levels of Bax and Bcl-2 proteins in both GFP-positive and -negative cells. As shown in Fig. 3b, we observed significant differences in the expression of Bax and Bcl-2 between the two cell populations. In particular, in GFP-positive cells we observed a significant reduction of Bax and a mild increase of Bcl-2 levels, with a consequent decrease of Bax/Bcl-2 ratio in comparison to GFP-negative cells (Fig. 3b). Consistently, we detected a significant (*p* < 0.01) reduction of CHX+TNF-α-induced apoptosis levels in GFP-positive vs GFP-negative cells (Fig. 3c). In Fig. S2 the analyses obtained in a second, independent round of experiments using DFs isolated from three additional male subjects are reported. In few words, the overexpression of miR548am-5p in XY cells leads to an increased resistance to apoptotic cell death, thus reverting the natural proneness of XY cells to undergo apoptosis.

### Effects of down-regulation of miR548am-5p in XX DFs

**Fig. 4:**
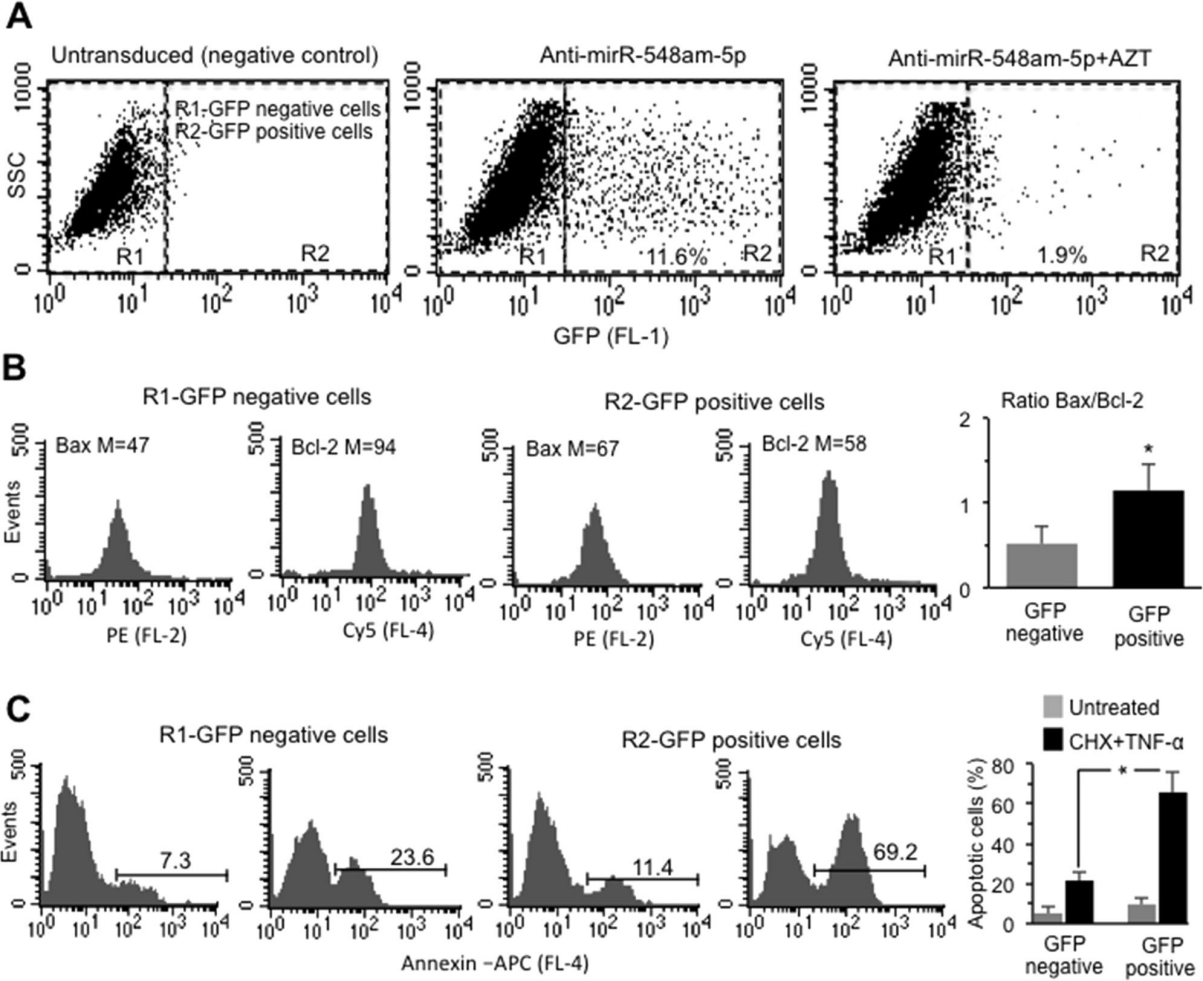
**a** Side scatter vs (FL1) GFP dot plots obtained in one representative XX DFs cell line untransduced or transduced with GFP-anti-miR548am-5p in the presence or absence of AZT. Numbers indicate the percentage of GFP-positive cells. **b** Cytofluorimetric histograms of Bax and Bcl-2 expression level in GFP-negative and -positive cells obtained in one representative XX DFs cell line after transduction with GFP-anti-miR548am-5p. Numbers indicate the median fluorescence intensity. Bar graph on the right shows the ratio Bax/Bcl-2 of four XX DFs cell lines. *p < 0.01 between GFP-positive and GFP-negative cells. **c** Flow cytometry histograms of apoptosis evaluation in GFP-positive or -negative cells of one representative XY DFs cell line after transduction with GFP-anti-miR548am-5p treated or not with CHX+TNF-α. Numbers represent the percentages of Annexin V-positive cells. Bar graph showing flow cytometry analysis after cell staining with Annexin V-APC performed in triplicate and reported as means ± SD of four XX DFs cell lines

On the basis of the above results, the “reverse” experiment was then carried out: miR-548am-5p was down-regulated by transduction with anti-miR-548am-5p-carrying lentiviral construct containing GFP in DFs isolated from four female donors. These experiments were carried out in cells pre-treated or not with AZT. Consistently with previous reports^43^, transduction efficiency of XX DFs was significantly lower than that observed in XY ones (10.5 ± 1.8; Fig. 4a, central panel, Fig. S1C).

Forty-eight hours after transduction, GFP-positive cells were sorted by FACS and the level of miR548am-5p, quantitated by qRT-PCR, decreased by 55% compared to the GFP-negative controls (Fig. S1D).

Consistently with the reduced level of the miR548am-5p, the expression of the target protein Bax resulted increased whereas Bcl-2 expression was reduced in GFP-positive XX DFs. As a consequence, the Bax/Bcl-2 ratio increased significantly in transduced cells (Fig. 4b). Accordingly, a significant increase of apoptosis in transduced XX DFs upon treatment with CHX+TNF-α was observed (Fig. 4c). Also, in XX DFs, transduction per se induced a certain cytotoxic effect, as shown in Fig. 4c. Figure S3 shows the analyses obtained in a second, independent round of experiments using DFs isolated from three additional female subjects. In few words, the down-regulation of miR548am-5p in XX cells leads to an increased susceptibility to apoptotic cell death, reverting the natural attitude of XX cells to be resistant to apoptotic stimulus.

## Discussion

Sexual dimorphism is ubiquitous across vertebrates, in which both sexes exhibit differences in behavioral, morphological, and physiological trait. There is still some debate, but it is accepted that natural selection and/or sexual selection are important for the appearance of sexual dimorphism^44^, in which ecological and environmental factors may induce dimorphic traits in females and males harboring highly similar genome. In fact, most of the phenotypic differences are caused by a sex-biased expression of genes^45^, which can depend on their regulation at the transcriptional and post-transcriptional levels, and regulators such as miRs are reported to have important roles in this process^46^.

MiRs are endogenous small non-coding RNAs with a total length of about 19–25 nucleotides that exert a wide range of biological functions in different cellular processes, including differentiation, proliferation, metabolism, and apoptosis. MiRs can act at post-transcriptional level by inhibiting the translation of target mRNAs or directly stimulating mRNA degradation via binding the 3′ untranslated region (UTR) of target genes^47^, and it is believed that more than 30% of human genes are regulated by miRs.

Recent studies suggested that sex-related differences are present in the control of cell death programs^48^. As a general rule, XY and XX cells appear differently responsive to injuries possibly because of their different capabilities to face cellular stress^49^. Accordingly, it was observed that the same stressors preferentially induce apoptosis in XY cells and autophagy in XX cells, which can lead to cell survival^38,50,51^. Interestingly, this phenomenon has been observed not only in transformed cell lines but also in non-transformed cells freshly isolated from male or female human or animal tissues, e.g., human fibroblasts^52^, endothelial and smooth muscle cells, and rat microvascular cells. This different susceptibility has been connected to the content of reduced glutathione, which resulted more elevated in XX cells^37,53,54^. Another possible reason is the differential expression of regulatory elements such as miRs. A number of miRs is reported to be differentially expressed between men and women^24^. In this study, eight different datasets of miR expression were investigated and identified 73 female-biased miRNAs and 163 male-biased miRNAs across four tissues. These datasets were generated from miR expression analyses. We used a different approach, based on an *a priori* identification of miRs based on database exploration. In order to provide a proof of principle of the validity of this method, we investigated the expression of X-linked miRs in normal DFs from male and females subjects. We have found a number of miRs that are associated to XCI escaper genes. One of these miRs, miR-548-am-5p, resulted in fact much more expressed in XX cells. This miR is involved in the regulation of genes, among others, that are part of the P53 pathway, *i.e*. Bax, which represents a key actor in mitochondrial-mediated apoptosis^55^. Consistently, and in agreement with our previous results, the susceptibility to apoptosis resulted much higher in XY DFs than in XX DFs.

In the mitochondrial pathway of apoptosis, various types of stressors may cause translocation of pro-apoptotic proteins of the Bcl-2 family, *e.g*. Bax from the cytosol to the mitochondria where it can undergo oligomerization, resulting in increased mitochondrial permeability, release of cytochrome *c* from the mitochondria, and, finally, apoptosis execution^56^. The expression of this gene is regulated by the tumor suppressor P53 and has been shown to be involved in P53-mediated apoptosis. Thus, it is reasonable to assume that the higher Bax/Bcl-2 ratio found in XY DFs, compared to XX DFs, could be responsible for, or contribute to, the different apoptotic proneness observed between XY and XX DFs by favoring Bax oligomerization, release of cytochrome *c* from the mitochondria with a consequent mitochondrial membrane depolarization and, ultimately, apoptosis. On the basis of our results, we can state that miR548am-5p has a significant role in this sex-based difference in apoptosis susceptibility observed in DFs. In fact, in XY DFs transduced with miR548am-5p, we observed a significantly reduced Bax/Bcl-2 ratio and an increased resistance to CHX+TNF-α-induced apoptosis. Conversely, in XX DFs transduced with anti-miR548am-5p we observed a significant increase of both Bax/Bcl-2 ratio and susceptibility to CHX+TNF-α-induced apoptosis.

Very interestingly, the diverse transduction efficiency we observed between male and female DFs is consistent with other reports that showed reduced transduction efficiency of female cells and animals with viral vectors^43^. Although this evidence was not discussed previously, in the present study, we can hypothesize that the expression of VSV-G receptor (used as cell entry molecule) is expressed at lower titer on XX cells. Alternatively, given the reported evidence that female cells develop a more intense innate immune response than male cells^57^, it is conceivable that the lentivirus replication is contrasted in XX DFs resulting in a reduced number of GFP-positive cells.

It has been suggested that genes escaping XCI may account for the differential sensitivity of females to some diseases. For example, the inactive X chromosome frequently reactivates in cancers, mainly in breast cancer, and some of these sleeping genes can reawake as women age. We provide here a further actor in this scenario: the XCI-escaping miRs. In fact, our results demonstrate that the sex-specific susceptibility to apoptosis of DFs can be explained, at least in part, by the differential expression level and activity of a XCI-escaping miR, the miR-548am-5p. A number of questions remain open, including the importance of this phenomenon for tissues and cell types other than DFs, and the biological interaction between miR548am-5p and its BCL2 and Bax mRNA targets. Further studies are needed to clarify these important points; however, these data may pave the way for a new perspective on sex-specific susceptibility (or resistance) to the onset of many diseases as well as on sex-specific diseases prevention and treatment.

## Supplementary information

Supplmentary Figure S1

Supplmentary Figure S2

Supplementary Figure S3

legends of supplemenatal figures

Supplementary tables
