# Correction: X-chromosome-linked miR548am-5p is a key regulator of sex disparity in the susceptibility to mitochondria-mediated apoptosis

**DOI:** 10.1038/s41419-019-2069-0

**Published:** 2019-11-04

**Authors:** Paola Matarrese, Paolo Tieri, Simona Anticoli, Barbara Ascione, Maria Conte, Claudio Franceschi, Walter Malorni, Stefano Salvioli, Anna Ruggieri

**Affiliations:** 1 Center for Gender Specific Medicine, Istituto Superiore di Sanità, viale Regina Elena 299, Rome, Italy; 2 CNR National Research Council, IAC Institute for Applied Computing, Via dei Taurini 19, Rome, Italy; 3 Data Science Program, La Sapienza University of Rome, Rome, Italy; 4 Department of Experimental, Diagnostic and Specialty Medicine (DIMES), University of Bologna, Bologna, Italy; 5 Interdepartmental Centre “L. Galvani” (CIG), University of Bologna, via San Giacomo 12, 40126 Bologna, Italy; 6 IRCCS Istituto delle Scienze Neurologiche di Bologna, Via Altura 3, 40139 Bologna, Italy; 7 Lobachevsky State University of Nizhny Novgorod, Nizhny Novgorod, Russia; 8 School of Mathematical, Physical and Natural Sciences and Faculty of Medicine, University of Tor Vergata, Rome, Italy

**Keywords:** Apoptosis, Genetics research

**Correction to: Cell Death & Disease**

10.1038/s41419-019-1888-3, published online 11 September 2019.

Since online publication of this article, the authors noticed that the Supplementary Tables S1–S8 were absent from the published version. The article has now been corrected to include these. The authors apologize for any inconvenience caused.

