# Supplementary figures and images for: X-chromosome-linked miR548am-5p is a key regulator of sex disparity in the susceptibility to mitochondria-mediated apoptosis
Source: Cell Death Dis. 2019 Sep 11;10(9):673. doi: 10.1038/s41419-019-1888-3 (PMC6739406; doi:10.1038/s41419-019-1888-3)

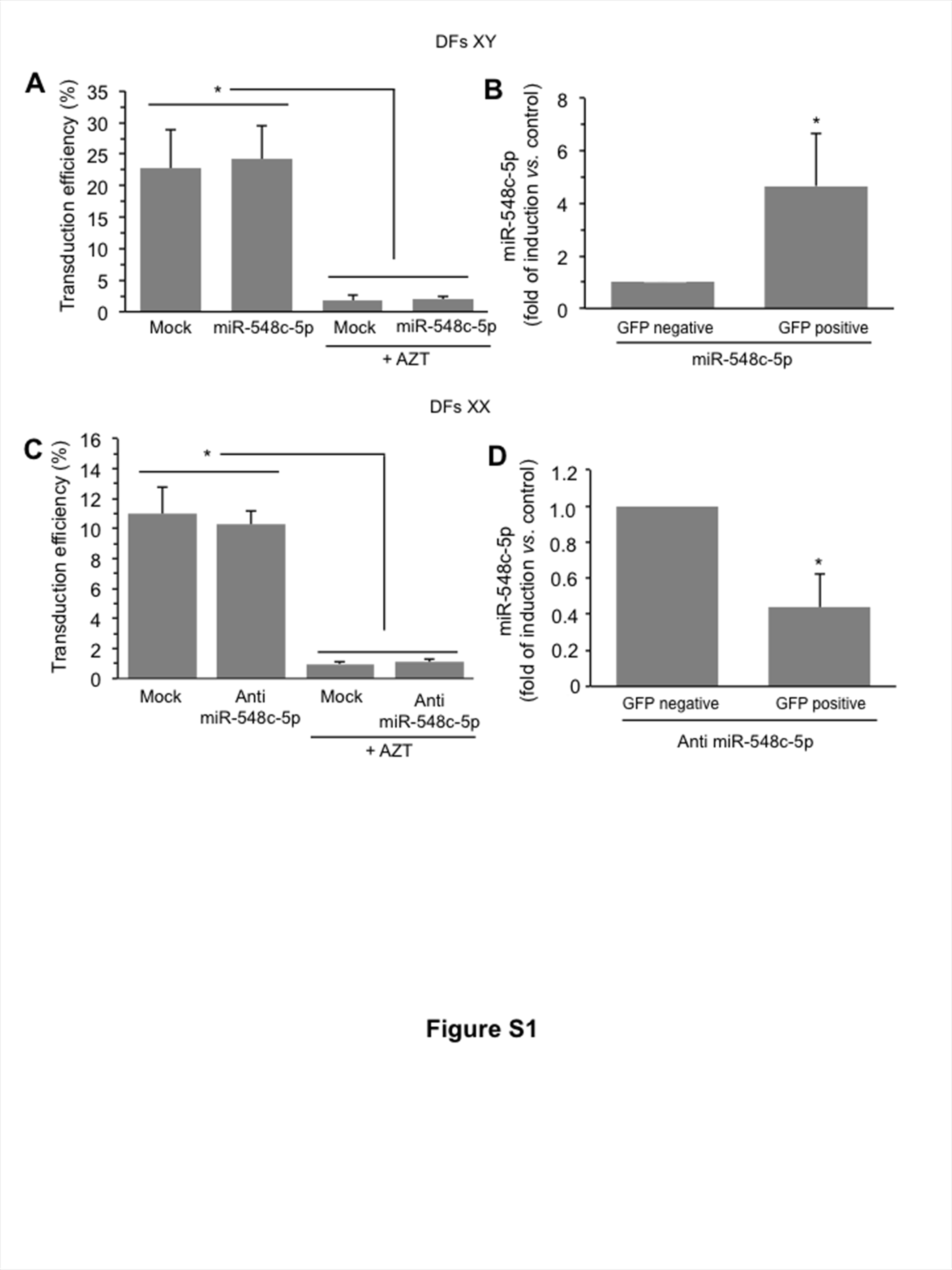

Supplement: Supplementary file 1 — Supplmentary Figure S1 [file 41419_2019_1888_MOESM1_ESM.tif]

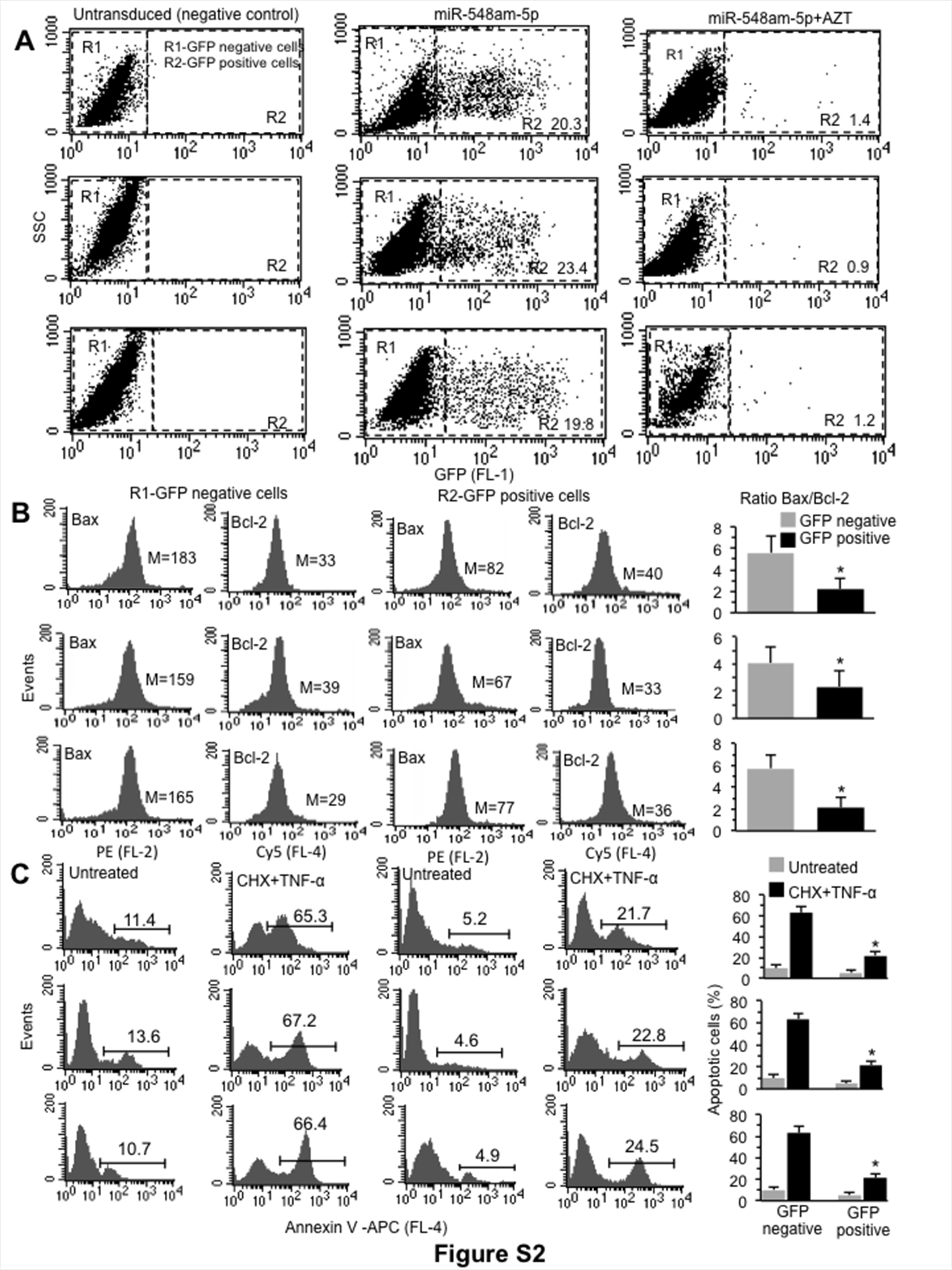

Supplement: Supplementary file 2 — Supplmentary Figure S2 [file 41419_2019_1888_MOESM2_ESM.tif]

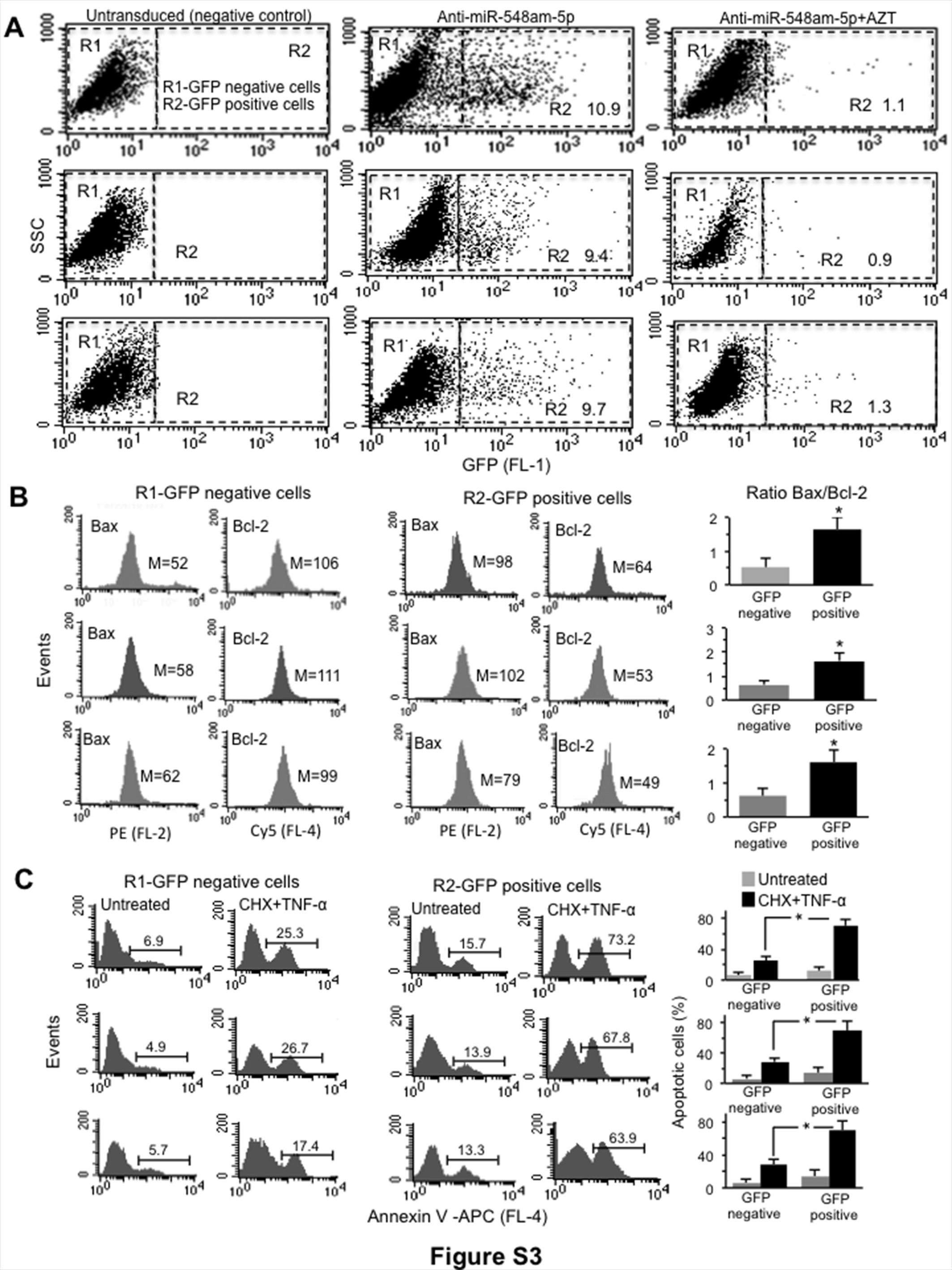

Supplement: Supplementary file 3 — Supplementary Figure S3 [file 41419_2019_1888_MOESM3_ESM.tif]
