# Supplementary material for: X-chromosome-linked miR548am-5p is a key regulator of sex disparity in the susceptibility to mitochondria-mediated apoptosis
Source: Cell Death Dis. 2019 Sep 11;10(9):673. doi: 10.1038/s41419-019-1888-3 (PMC6739406; doi:10.1038/s41419-019-1888-3)
Supplement: Supplementary file 4 — legends of supplemenatal figures [file 41419_2019_1888_MOESM4_ESM.docx]

**Supplementary Figure S1**. (**A**) Transduction efficiency of DFs isolated from male subjects. Numbers indicate the percentage of GFP-positive cells. (**B**) Expression level of mir548am-5p in XY DFs quantitatively measured by qRT-PCR. (**C**) Transduction efficiency of DFs isolated from female subjects. Numbers indicate the percentage of GFP-positive cells. (**D**) Expression level of mir548am-5p in XX DFs quantitatively measured by qRT-PCR.

The values of fold increase were calculated by the 2^-deltadeltaCt method for each sample relative to the GFP-negative control mean. The fold increase values were obtained from four male and four female donors estimated in two independent quantitation reactions, with triplicate wells for each sample.

**Supplementary Figure S2**. (**A**) Side scatter *versus* GFP (FL1) dot plots obtained in three representative XY DFs cell lines untransduced or transduced with GFP-miR548am-5p in the presence or absence of AZT. Numbers indicate the percentage of GFP-positive cells. (**B**) Cytofluorimetric histograms of Bax and Bcl-2 expression level in GFP-negative and positive cells obtained in three representative XY DFs cell lines after transduction with GFP-miR548am-5p. Numbers indicate the median fluorescence intensity. Bar graph on the right shows the ratio Bax/Bcl-2. (**C**) Flow cytometry histograms of apoptosis evaluation in GFP-positive or negative cells of three representative XY DFs cell lines after transduction with GFP-miR548am-5p treated or not with CHX+TNF-α. Numbers represent the percentages of Annexin V-positive cells. On the right, bar graph showing flow cytometry analysis after cell staining with Annexin V-APC performed in triplicate and reported as means ± SD. (*) Indicates p<0.01 between GFP-positive and GFP negative cells.

**Supplementary Figure S3**. (**A**) Side scatter *versus* GFP (FL1) dot plots obtained in three representative XX DFs cell lines untransduced or transduced with GFP-anti-miR548am-5p in the presence or absence of AZT. Numbers indicate the percentage of GFP-positive cells. (**B**) Cytofluorimetric histograms of Bax and Bcl-2 expression level in GFP-negative and positive cells obtained in three representative XX DFs cell lines after transduction with GFP-anti-miR548am-5p. Numbers indicate the median fluorescence intensity. Bar graph on the right shows the ratio Bax/Bcl-2. (**C**) Flow cytometry histograms of apoptosis evaluation in GFP-positive or negative cells of three representative XX DFs cell lines after transduction with GFP-anti-miR548am-5p treated or not with CHX+TNF-α. Numbers represent the percentages of Annexin V-positive cells. On the right, bar graph showing flow cytometry analysis after cell staining with Annexin V-APC performed in triplicate and reported as means ± SD. (*) Indicates p<0.01 between GFP-positive and GFP negative cells.
